# A new look at cerebrospinal fluid movement

**DOI:** 10.1186/2045-8118-11-16

**Published:** 2014-07-27

**Authors:** Darko Orešković, Marijan Klarica

**Affiliations:** 1 Ruđer Bošković Institute, Department of Molecular Biology, Bijenička 54, 10 000 Zagreb, Croatia; 2 Department of Pharmacology and Croatian Institute for Brain Research, School of Medicine University of Zagreb, Zagreb, Croatia

**Keywords:** Classic hypothesis of cerebrospinal physiology, Cerebrospinal fluid circulation, Cerebrospinal fluid movement, Cerebrospinal fluid secretion, Aquaporin, Orešković and Klarica hypothesis

## Abstract

Brinker *et al.* extensively reviewed recent findings about CSF circulation in a recent article: “A new look at cerebrospinal circulation”, but did not analyze some important available data in sufficient detail. For example, our findings as well as some clinical data and experimental results obtained from different animal species, do not support unidirectional CSF circulation but strongly suggest that there are cardiac cycle-dependent systolic-diastolic to-and-fro cranio-spinal CSF movements. These are based on: a) physiological oscillations of arterial and venous blood during cranio-spinal blood circulation; b) respiratory activity, and c) body activity and posture. That kind of complex CSF movement could explain the observed distribution of many different substances in all directions along the CSF system and within central nervous system tissue. It seems that efflux transport systems at capillary endothelium may be more important for brain homeostasis than the removal of metabolites by CSF flow. Thus, when discussing the CSF dynamics we suggest that a more appropriate term would be CSF movement rather than CSF circulation.

## Background

Brinker *et al.*[1] have described some recent views on cerebrospinal fluid (CSF) circulation. They nicely summarized current achievements in the CSF physiology which open new vistas, and stressed the need of reconsidering the traditional hypothesis of CSF physiology. Namely, according to the “classic” hypothesis, CSF is mainly produced by the choroid plexuses inside brain ventricles, then it circulates unidirectionally through the ventricles, and further along the subarachnoid spaces (SAS), to be passively absorbed into the venous blood across arachnoid villi. However, numerous findings on experimental animals, as well as novel insights obtained by molecular, cellular and neuroimaging approaches indicate that CSF physiology is, in fact, much more complex. Although Brinker *et al.* concur with our long-standing argument for developing new approaches to CSF physiology, they, unfortunately, failed to correctly interpret some of our published results. Thus, they state that: *For example, Klarica et al. failed to reproduce the historical experiments of Dandy*[2]*, since no circulation of CSF was found along a plastic cannula introduced into the aqueduct of cats*[3]*. Subsequent experiments demonstrated that the CSF pressure is not increased during first hours after the occlusion of aqueduct of Sylvius*[4]*. Since they furthermore showed that following its intraventricular injection radioactive water is almost completely absorbed in the ventricles and does not reach the basal cisterns*[5]*, they concluded that choroid plexus is not the major site of CSF production and that no direct CSF circulation according classical understanding exists. Instead they proposed a model that assumes CSF production and absorption occurs at the level of the capillaries*[6]*. Furthermore the view of Klarica et al. that CSF production and absorption just depend on hydrodynamic and osmotic gradients is not substantiated by current cellular and molecular biology findings. In fact, the proposed model does not consider the complex regulation of water movement between the brain compartments as discussed above. Finally, as in the original experiments of Dandy, the experiments of Klarica et al. may be criticized since they are surgically invasive and therefore results should be interpreted cautiously”.*

## Discussion

We did not attempt to reproduce the experiment of Dandy [2]. Quite the opposite: we developed several original experimental models in both cats and dogs to test the basic tenets of the “classic” CSF hypothesis [3-10]. While some of our models were indeed based on neurosurgical treatment [3,7,9], the majority of our experiments were based on freely-moving dogs and cats [5,8,10]. In both cases, our findings could not be fitted into “classic” CSF physiology hypothesis. Furthermore, our recent experiments in anaesthetized cats involved no surgical intervention apart from invasive CSF pressure monitoring [11]. We observed the hydrostatic pressure gradient in the upright position and found a long-lasting sub-atmospheric intracranial pressure, zero CSF pressure in the cervical region and +30 cm H_2_O in the lumbar region. This does not support the traditional CSF “circulation” along the cranio-spinal CSF space because CSF can only flow from a region of higher to a region of lower CSF pressure [11].

Based on our experimental results and literature data, we proposed a new hypothesis (“model”) of CSF physiology [6]. We wish to emphasize that our hypothesis is based on more than thirty years of continuous experimental research. According to our hypothesis, CSF is not being actively produced predominantly by choroid plexuses, and does not flow unidirectionally to cortical SAS to be passively absorbed through arachnoid villi. This means that CSF is being permanently produced and absorbed inside the entire CSF system, as a consequence of water filtration and reabsorption through the capillary walls into the interstitial fluid (ISF) of the surrounding brain tissue, which was thoroughly explained in our paper [5,6,12]. In brief, it was demonstrated that water passage between cerebral capillaries and ISF is relatively free [13,14], while the passage of proteins and inorganic electrolytes is greatly restricted. Our results obtained on cats show that during slow infusion into the lateral ventricle, ^3^H-water reached a concentration several times higher in the plasma at the confluence of the sinuses than that in cisternal CSF and arterial plasma [5]. Thus, it appears that ^3^H-water is absorbed from the brain ventricles into periventricular capillaries which drain into the confluence.

It is true that our research has not been conducted at the molecular or cellular level, but that is because we were investigating this phenomenon at the level of systems physiology [3-11]. However, our hypothesis has been strongly supported and our investigation extended as in a recent molecular study of Nakada’s research team [15], who analyzed water passage from the brain into the CSF in aquaporin-1 (AQP-1), aquaporin-4 knockout and wild-type mice using a newly-developed water molecular MRI technique. They concluded [15] that: *“…water flux into CSF in AQP-1, AQP-4 knockout mice utilizing H*_*2*_^*17*^*O JJVCPE imaging to test the hypothesis that water movement within the pericapillary (Virchow–Robin) space is critical for CSF volume homeostasis (Orešković and Klarica hypothesis). The result clearly demonstrated that water influx into CSF is regulated by AQP-4, known to be responsible for water homeostasis of the pericapillary space, and not by AQP-1 found in the choroid plexus, strongly supporting the Orešković and Klarica hypothesis”.*

The subarachnoid CSF and nervous parenchyma are separated from each other by a layer of thin pial cells without specialized intercellular junctions [16], so that the exchange of substances across the pial layer is not significantly restricted [8,12]. The Virchow-Robin paravascular space, although small in size (virtual space) [17], enables a rapid distribution of large molecules from the CSF system into the brain parenchyma and *vice-versa*, as was repeatedly shown in many publications [18-20]. Evidence for paravascular fluid movement in the mammalian central nervous system (CNS) is provided by the rapid distribution of tracer protein throughout the brain from the subarachnoid space [18].

But if there is no unidirectional CSF “circulation”, as is proposed by our hypothesis, in what way would one describe CSF dynamics? Since the CSF is 99% water, it would be logical to expect that dynamics of water should represent the dynamics of CSF, because, by definition, circulation is a unidirectional flow of volume. However, CSF/water volume is slowly carried bidirectionally along all CSF spaces by the systolic-diastolic to-and-fro displacement [3,10]. This movement of fluid is different in the spinal, compared to the cranial subdural space, due to different anatomical and biophysical characteristics of the cranial and spinal compartments [10,11,21-24]. Namely in the cranium, the dura is firmly attached to the bone and the subdural volume cannot not be changed significantly. On the other hand, the spinal dura is only partially attached to the vertebrae and is separated from the bone by an epidural space filled with venous plexus and fatty tissue. So it was observed that the lumbar space can be significantly altered in different physiological states (hyperventilation, hypoventilation, pressure applied to the abdomen) [23]. These changes in cervical or lumbar space volume result in adaptive filling and emptying of the venous plexuses in response [11,21,22].

All other substances in CSF (1% of total CSF volume) are distributed in all directions throughout the entire CSF system, and between CSF and CNS tissue, i.e. in the direction of “circulation” (out of the ventricles), as well as in the opposite direction (from SAS into the ventricles) [5,6,8,10]. The distribution distance of any substance within the CSF space depends on the half-life of this substance inside the CSF. Our results suggest that efflux transport at the capillary endothelium is much more important for brain homeostasis than the removal of potential toxic brain metabolites by CSF “circulation” [8,10]. Substances that are being removed more slowly from the CSF and CNS tissue, will eventually reach a greater distance. Further, our results from studies on dogs and cats in control groups show two striking phenomena: a) a fast disappearance of organic acids from the central CSF compartment (cisterna magna, cisterna basalis), and b) the very low concentration of tested substances in “peripheral” CSF compartments (cortical and lumbar subarachnoid space) [8,10]. However, when the active transport was blocked in animals by probenecid, the concentration of monitored substances in parenchyma and in “peripheral” CSF compartments [8,10] was significantly increased (during the same period of time) in comparison to control animals.

Therefore, in interpretation of experimental results it is very important to distinguish the movement of CSF/water on one hand, and the distribution of substances inside the CSF system on the other. The main misunderstanding relating to the study of CSF “circulation” has been made by (mis)interpretation of experimental results in which the two physiological processes (movement of CSF and distribution of substances) have not been differentiated, and when substance distribution has been used as a marker of “circulation”. Namely, the monitoring of certain substances in CSF system will reveal nothing about the “circulation” of CSF, but only about the distribution of that substance within the CSF.

## Conclusion

When discussing the dynamics of CSF, the term “circulation” of CSF should be avoided, and a more appropriate term “movement” of CSF would be advisable. Also, there is no sensible experimental evidence to consider CSF as a third circulation.

## Abbreviations

AQO: Aquaporin; BBB: Blood brain barriers; CNS: Central nervous system; CSF: Cerebrospinal fluid; ISF: Interstitial fluid; SAS: Subarachnoid space.

## Competing interest

The authors declare that they have no competing interest.

## Authors’ contributions

Both authors contributed equally to this work and have read and approved the final version of the manuscript.
